# *Coptis japonica Makino* ethanol extracts attenuates cancer cachexia induced muscle and fat wasting through inhibition of the STAT3 signaling pathway

**DOI:** 10.3389/fnut.2025.1509086

**Published:** 2025-05-21

**Authors:** Yo Sep Hwang, Eun Sun Park, Jahyeong Han, Suk Ran Yoon, Jun-Pil Jang, Jong Seok Lim, Seong-Hoon Park, Jun Hong Park, Hee Jun Cho, Hee Gu Lee

**Affiliations:** ^1^Immunotherapy Research Center, Korea Research Institute of Bioscience and Biotechnology, Daejeon, Republic of Korea; ^2^Department of Biomolecular Science, KRIBB School of Bioscience, Korea University of Science and Technology (UST), Daejeon, Republic of Korea; ^3^Chemical Biology Research Center, Korea Research Institute of Bioscience and Biotechnology, Cheongju, Republic of Korea; ^4^Department of Biological Science and the Cellular Heterogeneity Research Center, Research Institute of Women's Health, Sookmyung Women's University, Seoul, Republic of Korea; ^5^Genetic and Epigenetic Toxicology Research Group, Korea Institute of Toxicology, Daejeon, Republic of Korea; ^6^Herbal Medicine Resources Research Center, Korea Institute of Oriental Medicine, Naju-si, Republic of Korea

**Keywords:** *Coptis japonica Makino*, cancer cachexia, muscle atrophy, STAT3, IL-6

## Abstract

Cancer cachexia is a complex syndrome marked by appetite loss, weakness, fatigue, significant weight loss, and depletion of both adipose and muscle tissue, driven by metabolic and inflammatory alterations caused by tumors. Cachexia is a critical contributor to poor cancer prognosis, often leading to reduced efficacy of treatments. *Coptis japonica Makino* (CJM) is a medicinal herb widely used in Asia, known for its anti-inflammatory and metabolic regulatory properties. However, its potential role in cancer cachexia has not yet been explored. This research aimed to explore the potential of CJM extracts (CJME) in mitigating cancer cachexia in both myotubes treated with CT26 conditioned medium (CM) and in a CT26-induced cancer cachexia mouse model. Our results demonstrated that CJME significantly decreased the mRNA and protein levels of muscle-specific E3 ubiquitin ligases Atrogin-1 and MuRF1 in myotubes exposed to CT26 CM. Furthermore, CJME notably enhanced the protein levels of myosin heavy chain (MyHC). In the mouse model of CT26-induced cancer cachexia, severe loss of muscle and fat was observed. however, CJME effectively countered this wasting and restored abnormal biochemical parameters such as CK, albumin, triglycerides (TG), cholesterol, high-density lipoprotein (HDL), and low-density lipoprotein (LDL) associated with cancer cachexia. Moreover, CJME reduced interleukin-6 (IL-6) levels in both CT26 CM-stimulated myotubes and the serum of CT26-induced cancer cachexia mice. The mechanism underlying these effects appears to involve the suppression of STAT3 activation by CJME. These findings suggest that CJME has potential as a therapeutic candidate in the management of cancer cachexia.

## 1 Introduction

Cancer cachexia is a multifaceted syndrome characterized by severe weight loss, significant muscle and fat depletion, profound fatigue, and a marked loss of appetite. This condition arises as a direct consequence of tumor proliferation, which induces alterations in metabolic pathways and leads to excessive secretion of inflammatory cytokines in cancer patients (1, 2). As tumors progress, they disrupt normal metabolic processes of the cancer patient, resulting in reduced appetite and the catabolism of both muscle and adipose tissue. This results in persistent weight loss, along with a reduction in both muscle and fat mass. Cancer cachexia critically impacts patient survival rates and overall quality of life. Cachexia is often associated with diminished responses to cancer therapies, further contributing to its role as a leading cause of mortality among cancer patients. Notably, in colorectal cancer, cachexia is observed in ~50–61% of patients, highlighting its prevalence and significance within this patient population (3, 4). Therefore, treating cancer cachexia is crucial for improving overall cancer treatment outcomes, as it not only enhances patients' quality of life but also optimizes their responses to therapeutic interventions.

Muscle atrophy in cancer cachexia is a complex process characterized by the degradation of skeletal muscle tissue, primarily driven by specific ubiquitin ligases such as muscle atrophy F-box 1 (MAFbx, also known as Atrogin-1) and muscle ring finger protein 1 (MuRF1) (5, 6). These proteins play a pivotal role in the muscle wasting observed in cancer patients by promoting the breakdown of muscle fibers. During this process, a range of critical muscle structural proteins including myosin heavy chains, myosin-binding protein C, and myosin light chains are targeted for ubiquitination by the ubiquitin-proteasome system. This post-translational modification promotes the degradation of these proteins, leading to a significant loss of muscle integrity and function (7, 8). The accumulation of these ubiquitinated proteins in the skeletal muscle of cancer patients serves as biomarkers for muscle wasting, indicating the severity of cachexia (9, 10). The activity of these muscle atrophy-related proteins is intricately regulated by various signaling pathways, including the STAT3, NF-κB, and MAPK pathways (11–13). These pathways are activated by inflammatory cytokines and other tumor-derived factors, which further exacerbate muscle degradation. The interplay between these signaling mechanisms not only facilitates the process of muscle atrophy but also highlights potential therapeutic targets for mitigating cachexia.

Blood biochemical markers, including creatine kinase (CK) and albumin, can be used to assess skeletal muscle damage. Serum CK levels not only reflect muscle loss but also the extent of muscle damage (14). Serum albumin levels are known to decrease in patients with cancer cachexia (15). Additionally, triglycerides (TG) hydrolysis is considered a crucial metabolic pathway involved in the initiation and progression of cachexia (16). In cachexia patients, total cholesterol, high-density lipoprotein (HDL), and low-density lipoprotein (LDL) levels often tend to decrease. This is due to weight loss, metabolic changes, and depletion of fat tissue affecting cholesterol production (17).

Blood biochemical markers such as creatine kinase (CK) and albumin are essential proteins for assessing skeletal muscle damage and nutritional status in affected patients. Elevated serum CK levels not only indicate muscle loss but also reflect the extent of muscle damage (14). Conversely, serum albumin levels typically decrease in patients suffering from cancer cachexia, reflecting the decline in nutritional status and overall health (15). Additionally, triglyceride (TG) hydrolysis plays a pivotal role in the metabolic disturbances associated with cachexia and influences the initiation and progression of this syndrome (16). In patients with cancer cachexia, lipid levels, including total cholesterol, high-density lipoprotein (HDL), and low-density lipoprotein (LDL), often tend to decrease. This reduction can be attributed to weight loss, metabolic changes, and the depletion of adipose tissue, which collectively interfere with cholesterol production (17). Understanding these biochemical changes is crucial for developing effective interventions and improving the quality of life for patients suffering from cancer cachexia.

*Coptis japonica Makino* (CJM) is a member of *Ranunculaceae* family and its rhizome has been used traditionally as herbal medicine in China and Japan for centuries. In traditional Asian medicine, CJM is known for its diverse therapeutic applications, including anti-inflammatory, antibacterial, and antipyretic properties, making it a significant herb in traditional medicine (18, 19). CJM extracts (CJME) has been reported to exhibit a range of beneficial effects including fungicidal activity against pathogens such as *Botrytis cineria, Erysiphe graminis, Phytophthora infestans, Puccinia recondita, Pyricularia grisea*, and *Rhizoctonia solani* (20). Additionally, CJME exhibits anti-angiogenic properties by inhibiting cell cycle-regulated proteins (21) and anti-photooxidative activity (22). Other noted effects include anti-inflammatory (23) and antibacterial actions (24), as well as peripheral nerve regeneration capabilities, evidenced by reduced neuroma and scar tissue formation in rat models (25). Despite these promising properties, studies on the potential role and regulatory mechanisms of CJME in cancer cachexia are still unknown.

In this study, we investigated the effects of CJME on muscle atrophy using a CT26 conditioned media system in C2C12 myoblast cells. We also assessed CJME's impact in a CT26-induced cancer cachexia mouse model. By exploring both *in vitro* and *in vivo* approaches, we aim to uncover how CJME may help mitigate the effects of cancer cachexia, offering new insights into its therapeutic potential for improving patient outcomes.

## 2 Materials and methods

### 2.1 Arrangements of CJM extracts

*Coptis japonica Makino* (CJM) extracts were prepared as previously reported (26). In summary, the lyophilized powder from a 70% ethanol extracts of CJM, supplied by KOC Biotech (KOC-70E-346, Daejeon, Korea), was reconstituted in 10% DMSO (D2650, Sigma, St. Louis, MO, USA), and which was filtered by 0.22 μm membrane.

### 2.2 Reagents and antibodies

The Myosin Heavy Chain antibody was acquired from R&D Systems (Minneapolis, MN, USA). Muscle-specific E3 ubiquitin ligases, Atrogin-1, muscle RING finger protein-1 (MuRF1), and β-actin antibodies were sourced from Santa Cruz Biotechnology (Dallas, TX, USA). Antibodies targeting p-STAT1, p-STAT2, p-STAT3, p-ERK, p-JNK, p-p38, p-IKKα/β, and p-IκBα were sourced from Cell Signaling Technology (CST, Danvers, MA, USA).

### 2.3 Cell culture and treatment

The murine myoblast cell line C2C12 and murine colon carcinoma cell line CT26 used in the experiment were sourced from ATCC (Rockville, MD, USA). The murine myoblast cell line C2C12 was cultured in DMEM (Gibco, NY, USA) supplemented with 10% FBS (HyClone, Logan, UT, USA) and antibiotics (Gibco). C2C12 myoblasts were differentiated for 2 days in medium containing 2% horse serum (Gibco). Differentiated C2C12 myotubes were pre-treated with CJME for 1 h before exposure to CT26 conditioned medium (CT26 CM) and cultured as indicated in each figure. The murine colon carcinoma cell line CT26 was cultured in RPMI 1640 medium (Gibco) supplemented with 10% FBS and antibiotics (Gibco). To generate CT26 conditioned medium, 5 × 10^6^ cells were seeded into a 150 mm culture dish (Corning, NY, USA), and the culture supernatant was harvested after 48 h. CT26 CM was applied to differentiated C2C12 myotubes at a 40% concentration.

### 2.4 Animal experiments

BALB/c mice (6–7 weeks old) were purchased from a local facility (DBL, Eumseong, Korea) and housed under controlled conditions in a SPF environment with a constant temperature of 23 ± 2°C, humidity of 50 ± 10%, and a 12-h light/dark cycle at the Animal Experiment Center of the Korea Research Institute of Bioscience and Biotechnology (KRIBB, Daejeon, Korea). CT26 cells (2.5 x 10^6^ cells per mouse) were subcutaneously injected into the flanks of the BALB/c mice. Starting from day 7 post-injection, CJME (10 and 20 mg/kg) dissolved in PBS (100 μL per mouse) was administered orally every 2 days for 3 weeks. The negative control group (Ctrl) did not receive CT26 cells, while the positive control group (CT26) was orally administered PBS (100 μL per mouse) at the same dose as CJME. Tumor width (W), depth (D), and height (H) were measured using a digital caliper, and the tumor volume was determined with the formula 0.52^*^(W^*^D^*^H). Four weeks after CT26 injection, the mice were sacrificed, and tumors were completely excised. The body weight of the mice was measured, and muscles including the pectoralis, triceps, quadriceps, tibialis anterior (TA), and gastrocnemius, along with adipose tissues epididymal white adipose tissue (eWAT), inguinal white adipose tissue (ingWAT), and interscapular brown adipose tissue (iBAT) were harvested, weighed, and rapidly frozen in liquid nitrogen for further analysis. All animal experiments were approved by the KRIBB Institutional Animal Care and Use Committee (KRIBB-AEC-21079).

### 2.5 Histology analysis

Differentiated C2C12 myotubes were washed with PBS, fixed with 4% paraformaldehyde (PAF, Sigma), and stained with 0.1% Crystal Violet solution (Sigma) for 10 min. After removing the staining solution, the cells were thoroughly washed with distilled water, followed by four additional washes with PBS. The stained myotubes were captured with a phase-contrast microscope (Zeiss). Myotube thickness was measured using ImageJ 1.53 software by averaging three measurements taken at different positions along each myotube. The gastrocnemius muscle and epididymal white adipose tissue (eWAT) were also fixed in 4% PFA, and paraffin sections were stained using hematoxylin and eosin (H&E). These stained sections were analyzed with microscopy. The areas of myofibers and eWAT were quantified with ImageJ 1.53 software. To create frequency distribution graphs, the areas of approximately 70–100 myofibers and adipocytes per field in each section were measured.

### 2.6 Blood sample collection and analysis

Blood samples were collected as previously described (26). In brief, CT26 cells (2.5 x 10^6^ cells per mouse) were subcutaneously injected into the flanks of BALB/c mice. Starting from day 7 post-injection, CJME (10 and 20 mg/kg) dissolved in PBS (100 μL per mouse) was administered orally once daily for 1 week. One week later, blood samples were collected via cardiac puncture following anesthesia with avertin (Sigma). The samples were centrifuged at 2,000 × g for 20 min to separate the serum, which was then stored at −80°C for subsequent analysis of creatine kinase (CK), albumin, cholesterol, triglycerides (TG), low-density lipoprotein (LDL), high-density lipoprotein (HDL), and cytokines including IL-1β, IL-6, and TNF-α. The levels of CK, albumin, TG, cholesterol, HDL, and LDL were measured with blood chemistry analyzer (Beckman Coulter, Krefeld, Germany).

### 2.7 Cell viability

Cell viability assay was performed as previously described (26). Briefly, The C2C12 myoblast cells were differentiated for 2 days in medium containing 2% horse serum (Gibco). Following differentiation, CJME treated into differentiated C2C12 for 48 h. Cell viability was assessed with the WST-1 assay (Roche, Pleasanton, CA, USA) following the manufacturer's instructions. Formazan production was calculated using a microplate reader (Molecular Devices, Sunnyvale, CA, USA).

### 2.8 Cytokines measurement

Cytokine levels were determined using DuoSet ELISA kits for mouse interleukin-1β (IL-1β, DY-401), interleukin-6 (IL-6, DY-406), and tumor necrosis factor-α (TNF-α, DY-410), all purchased from R&D Systems. The ELISA assays were performed in accordance with the standard protocols previously described (26).

### 2.9 Western blotting

The C2C12 myotubes were treated with RIPA buffer (Sigma) for lysis, and protein concentration was measured with a BCA assay (Intron Biotechnology, Seong-Nam, Korea). The protein samples of equal volume were loaded by 6–15% SDS-PAGE gel. After SDS-PAGE, the gels were transferred to PVDF membranes, which were then blocked with 5% skim milk and subsequently incubated using the following antibodies: MyHC (R&D, MAB4470), Atrogin-1 (SCBT, SC-166806), MuRF1 (SCBT, SC-398608), Phospho-STAT1 (CST, 9167), Phospho-STAT2 (CST, 4441), Phospho-STAT3 (CST, 9134), Phospho-ERK (CST, 9101), Phospho-JNK (CST, 9251), Phospho-p38 (CST, 9211), Phospho-IKKα/β (CST, 2697), Phospho-IκBα (CST, 2859), and β-actin (SCBT, SC-47778). After washing the primary antibody, the membranes were incubated with HRP-conjugated secondary antibodies for 1 h. The membranes were visualized using a chemiluminescent HRP substrate (Millipore, Billerica, MA, USA).

### 2.10 Quantitative real-time polymerase chain reaction analysis

Total RNA of cultured cells was extracted with Trizol RNA Isolation Reagents (Invitrogen, CA, USA) as previously described (26). Real-Time PCR was conducted with the QuantStudio 3 system (Thermo, Waltham, MA, USA). The mRNA expression levels of Atrogin-1 and MuRF1 were quantified in a 20 μL reaction volume that contained qPCR Master Mix (Bioneer, Daedeok-gu, Daejeon, Korea), in accordance with the manufacturer's guidelines. Relative mRNA levels were calculated with the 2–ΔΔCt method. Each sample was analyzed in duplicate, with a minimum of three samples per group. GAPDH expression (Ct value) did not show significant differences among all groups. The primers used were: Atrogin-1(F), 5′-ATGCAC ACTGGTGCAGAGAG-3′; Atrogin-1(R), 5′-TGTAAGCACACAGGCAGGTC-3′; MuRF1(F), 5′-GTCCATGTCTGGAGGTCGTT-3′; MuRF1(R), 5′-ACTGGAGCACTCCTGCTTGT-3′; GAPDH(F), 5′-ACCCAGAAGACTGTGGATGG-3′; and GAPDH(R), 5′-ACACATTG GGGGTAGGAACA-3′.

### 2.11 Liquid chromatography–mass spectrometry analysis

For the LC-MS analysis, we utilized an LTQXL linear ion trap mass spectrometer manufactured by Thermo Scientific (Rockford, IL, USA), which was equipped with an electrospray ionization (ESI) source. This configuration was paired with a rapid separation LC system (Ultimate 3000, Thermo Scientific) and a Waters HSS T3 column (Waters, Milford, MA, USA; 2.1 × 150 mm, 2.5 μm).

### 2.12 Statistical analysis

All statistical analyses were performed with GraphPad Prism 9 (San Diego, CA, USA). Data are presented as mean ± standard deviation and analyzed using a one-way ANOVA or Student's *t*-test. A *p* < 0.05 was deemed statistically significant, indicating meaningful differences between the groups.

## 3 Results

### 3.1 CJME attenuates CT26 CM-induced myotube atrophy in C2C12 cells by suppressing the STAT3 signaling pathway

**Figure 1:**
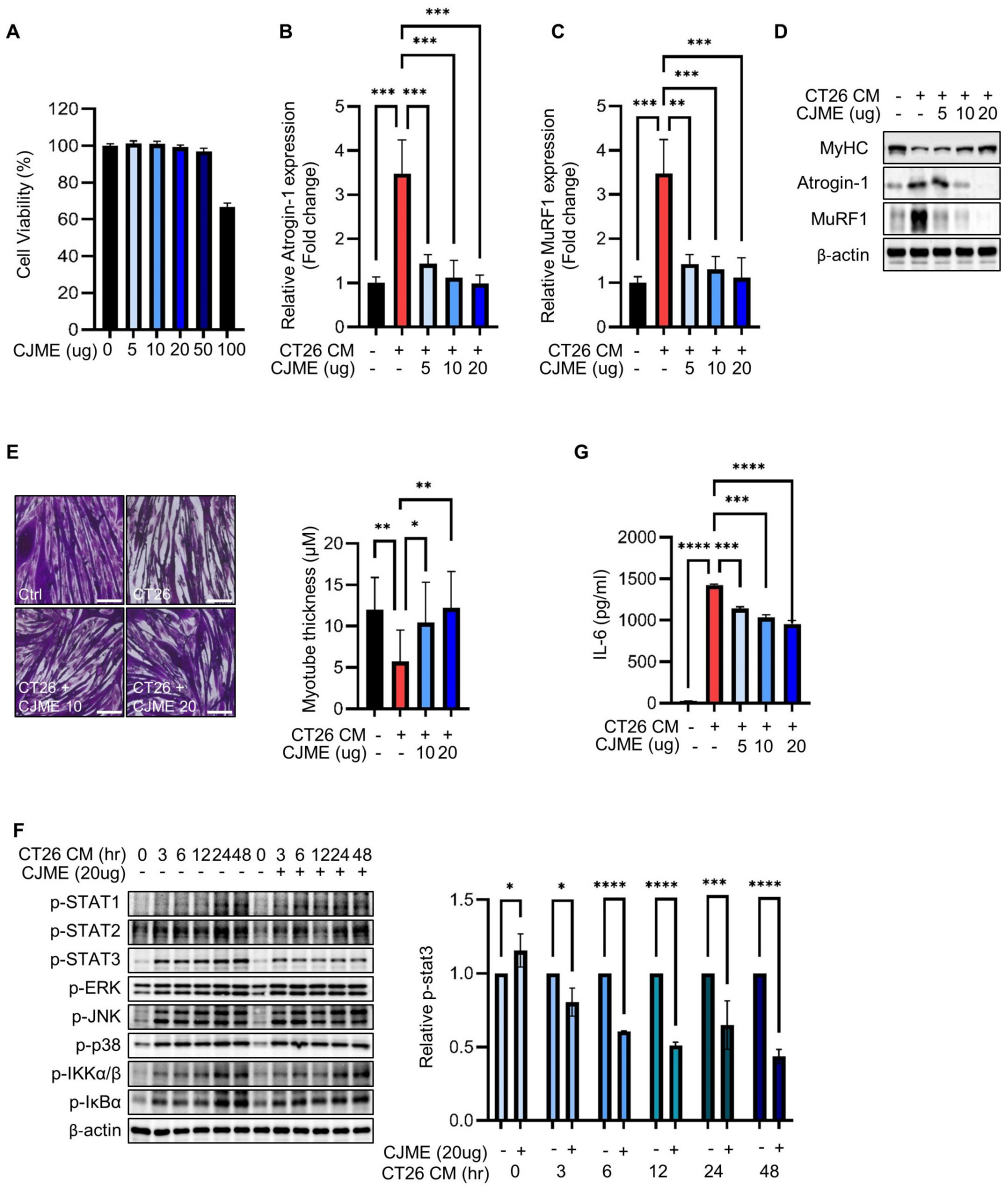
The effects of CJME on CT26 conditioned medium (CM) induced-myotube atrophy. The C2C12 myoblasts were differentiated for 2 days and induced to form myotubes. **(A)** Effect of CJME on differentiated myotubes viability were determined using WST-1 assay. **(B)** Indicated CJME concentrations were used for pretreatment 1 h prior to treatment with CT26 CM for 48 h in differentiated myotubes. mRNA levels of **(B)** Atrogin-1 and **(C)** MuRF1 were determined using real-time PCR. Relative mRNA expression levels were normalized to those of GAPDH. **(D)** Protein levels of MyHC, Atrogin-1 and MuRF1 were analyzed via immunoblotting in cells cultured as described in B. **(E)** Differentiated myotubes were cultured as described in B, fixed with 4% PAF and stained with Crystal violet. Representative images of stained myotubes were shown **(left)**. The scale bar represents 200 μm. Myotube thickness was measured using Image J 1.53 software **(right) (F)** Differentiated myotubes were pretreated with 20 μg/mL of CJME for 1 h and exposed to CT26 CM for various times (0, 3, 6, 12, 24, and 48 h). Then, phosphorylation of STAT1, STAT2, STAT3, ERK, JNK, p38, IKKα/β and IκBα was examined using immunoblotting **(left)**. Quantified data for STAT3 were measured using Image J 1.53 software **(right) (G)** Differentiated myotubes were cultured as described in B, secreted levels of IL-6 in the culture medium were measured using ELISA. Data are representative of either two independent experiments. Data are presented in terms of the mean ± standard deviation and analyzed using a one-wayANOVA or Student's *t*-test. *P* value of <0.05 (*), <0.01 (**), <0.001 (***), or <0.0001 (****) were considered statistically significant.

Differentiated C2C12 myotubes cells were treated with various concentrations of CJME (5, 10, 20, 50 and 100 μg/mL) for 48 h to assess cell viability. Although there was no significant effect on cell viability at concentrations up to 50 μg/mL, treatment with 100 μg/mL CJME reduced cell viability by approximately 40% (Figure 1A). Therefore, the CJME concentration for subsequent experiments was determined to be 5–50 μg/mL. Next, we treated C2C12 cells with conditioned media (CM) from CT26 colon cancer cells and evaluated the mRNA levels of muscle-specific E3 ubiquitin ligases, Atrogin-1 and MuRF1, which are key markers of skeletal muscle atrophy (6, 27). CT26 CM significantly elevated the mRNA levels of Atrogin-1 and MuRF1 in C2C12 myotubes, whereas CJME treatment effectively reduced the CT26 CM-induced increases in these mRNA levels (Figures 1B, C). Furthermore, we evaluated the protein expression of myosin heavy chain (MyHC), Atrogin-1, and MuRF1. Treatment with CT26 CM markedly increased the protein levels of Atrogin-1 and MuRF1 while decreasing MyHC expression. Notably, CJME treatment significantly reduced the CT26 CM-induced increases in Atrogin-1 and MuRF1 protein levels and restored MyHC expression (Figure 1D). Morphological analysis revealed that CT26 CM significantly reduced the thickness of C2C12 myotubes, an effect that was restored by CJME treatment (Figure 1E). To elucidate the signaling pathways by which CJME attenuates CT26 CM-induced myotube atrophy, we investigated the phosphorylation of key signaling proteins, such as STAT3, NF-κB, and MAPK. We found that CJME specifically inhibited the phosphorylation of STAT3 (Figure 1F), a critical regulator of skeletal muscle atrophy. Additionally, pro-inflammatory cytokines such as interleukin-6 (IL-6) and tumor necrosis factor-alpha (TNF-α) have been reported to be associated with muscle and fat wasting (28). In cancer cachexia, IL-6 is known to cause muscle and fat loss by activating the JAK-STAT signaling pathway (29, 30). Based on this, we measured IL-6 production in the culture medium of C2C12 myotubes. IL-6 production was significantly increased by CT26 CM, while treatment with CJME reduced the CT26 CM-induced increase in IL-6 production (Figure 1G). These findings suggest that CJME attenuates CT26 CM-induced myotube atrophy in C2C12 cells by suppressing the STAT3 signaling pathway and reducing pro-inflammatory cytokine production.

### 3.2 CJME protects against skeletal muscle loss in a CT26-induced cancer cachexia model

**Figure 2:**
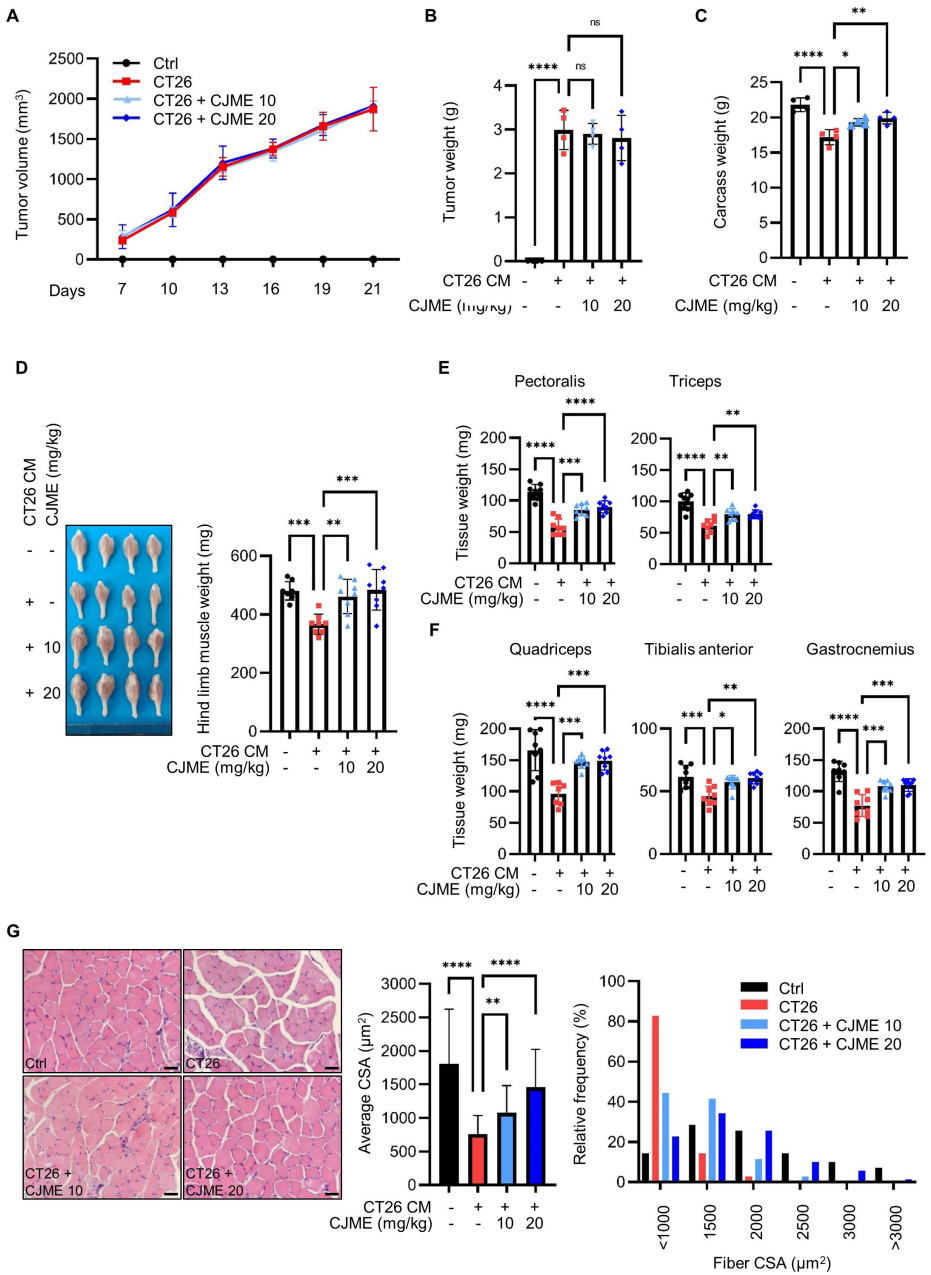
The effects of CJME on muscle wasting in CT26-induced cancer cachexia model. CT26 cells (2.5 × 10^6^/mouse) were subcutaneously injected into the flank of BALB/c mice, and starting from day 7 post-injection, CJME (10 and 20 mg/kg) was orally administered every 2 days for 3 weeks. The negative control group (Ctrl) was not injected with CT26 cells, and the positive control group (CT26) was orally administered PBS at the same dose as CJME. **(A)** Tumor volume was measured every 3 days starting from 7 days after the injection of CT26 cells. **(B)** After 4 weeks of CT26 cell injection, the tumors were excised, and tumor weight and **(C)** Carcass weight without the tumor mass were measured. **(D)** Representative images of the hind limb (Gastrocnemius, Soleus, Tibialis anterior muscle complex) were shown **(left)**, hind limb muscles weight was measured in CT26-induced cachexia mice model **(right)**. **(E)** Pectoralis and triceps muscle weight was measured in CT26-induced cachexia mice model. **(F)** Quadriceps, tibialis anterior (TA) and gastrocnemius muscle weight was measured in CT26-induced cachexia mice model. **(G)** Gastrocnemius muscle were stained with H&E staining, and representative images were shown **(left)**. The scale bar represents 50 μm. The average cross-sectional area (CSA) of gastrocnemius muscle fiber was quantified by Image J 1.53 software **(middle)** and fiber frequency distribution was quantified by Image J 1.53 software **(right)**. (*n* = 4 mice per group). Data are representative of either two independent experiments. Data are presented in terms of the mean ± standard deviation and analyzed using a one-wayANOVA or Student's *t*-test. *P* < 0.05 (*), <0.01 (**), <0.001 (***), or <0.0001 (****) were considered statistically significant.

Since the main characteristics of cancer cachexia patients are skeletal muscle wasting (31, 32), we investigated whether CJME could protect against skeletal muscle loss in a CT26-induced cancer cachexia mouse model. CT26 cells were subcutaneously injected into C57BL/6 mice. Starting from day 7 post-injection, CJME was orally administered every 2 days for 3 weeks, while the control group received an equal volume of PBS. There were no significant differences in tumor volume and weight between the groups (Figures 2A, B). However, the CT26-injected group that received PBS showed a significant decrease in body weight compared to the control group without CT26 injection (Ctrl). In contrast, the groups receiving CJME at 10 mg/kg (CT26+CJME 10) and 20 mg/kg (CT26+CJME 20) showed an increase in body weight compared to the CT26 group (Figure 2C). Additionally, the CT26 group exhibited a significant reduction in hind limb muscle weight compared to the Ctrl group, whereas both CJME-treated groups showed a significant increase in hind limb muscle weight compared to the CT26 group (Figure 2D). No significant difference was observed between the CT26+CJME 10 group and the CT26+CJME 20 group. We further investigated the effect of CJME on muscle loss in specific regions. Compared to the Ctrl group, the CT26 group showed reduced weights of the pectoralis, triceps, quadriceps, tibialis anterior (TA), and gastrocnemius muscles. In contrast, both the CJME-treated groups exhibited a significant increase in the weights of the pectoralis, triceps, quadriceps, TA, and gastrocnemius muscles compared to the CT26 group (Figures 2E, F). Furthermore, the relative muscle mass to body weight was also increased in the CJME-treated group compared to the CT26 group (Supplementary Table 1). Next, we performed H&E staining on gastrocnemius muscles and measured the cross-sectional area (CSA) of the gastrocnemius muscle fibers. The CSA of the gastrocnemius muscle was significantly smaller in the CT26 group compared to the Ctrl group. However, both the CJME-treated groups showed an increase in the CSA of the gastrocnemius muscle compared to the CT26 group (Figure 2G). Collectively, these results indicate that CT26-induced cancer cachexia in mice leads to muscle loss and dysfunction, and that CJME effectively reverses these effects.

### 3.3 CJME prevents fat tissue loss in CT26-induced cancer cachexia model

**Figure 3:**
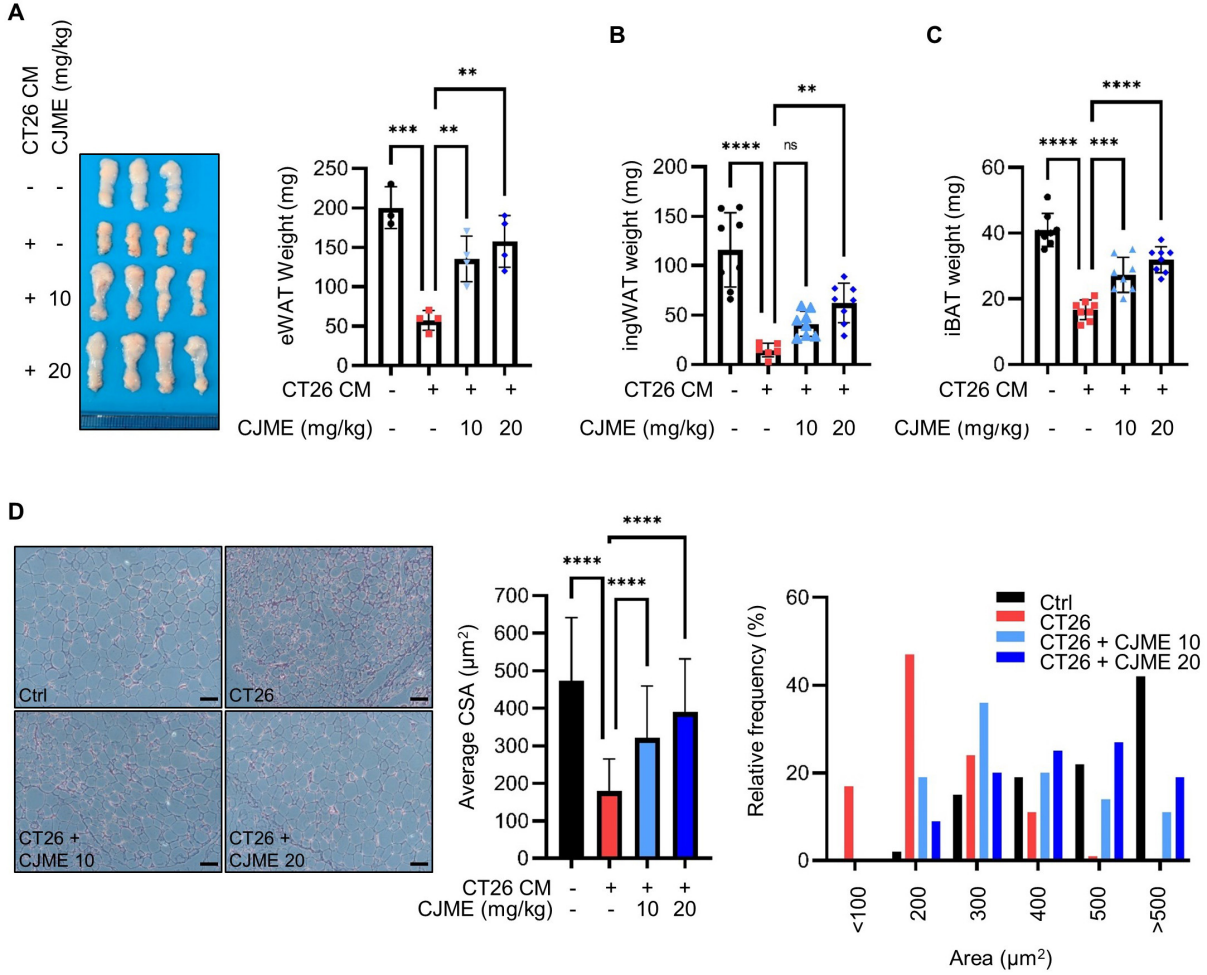
The effects of CJME on fat wasting in CT26-induced cancer cachexia model. CT26 cells (2.5 × 10^6^/mouse) were subcutaneously injected into the flank of BALB/c mice, and starting from day 7 post-injection, CJME (10 and 20 mg/kg) was orally administered every 2 days for 4 weeks. The negative control group (Ctrl) was not injected with CT26 cells, and the positive control group (CT26) was orally administered PBS at the same dose as CJME. **(A)** Representative images of the eWAT were shown **(left)**, eWAT weight was measured **(right)**. (*n* = 4 mice per group). **(B, C)** ingWAT and iBAT weight was measured. **(D)** eWAT was stained with H&E staining, and representative images were shown **(left)**. The scale bar represents 50 μm. The average cross-sectional area (CSA) of eWAT was quantified by Image J 1.53 software **(middle)** and frequency distribution of adipocyte cell area was quantified by Image J 1.53 software **(right)**. (*n* = 4 mice per group). Data are representative of either two independent experiments. Data are presented in terms of the mean ± standard deviation and analyzed using a one-wayANOVA or Student's *t*-test. *P* < 0.01 (**), <0.001 (***), or <0.0001 (****) were considered statistically significant.

Cancer cachexia is characterized by weight loss due to the loss of skeletal muscle and adipose tissue (33, 34). Therefore, we further investigated whether CJME could prevent not only muscle loss but also fat tissue loss in a CT26-induced cancer cachexia mouse model. Following the subcutaneous injection of CT26 cells into the flank of C57BL/6 mice, CJME was administered orally every 2 days starting from day 7 post-injection for a duration of 3 weeks. After the treatment, the mice were euthanized, and adipose tissue was harvested for further analysis. The weight of epididymal white adipose tissue (eWAT) was significantly reduced in the CT26 group compared to the Ctrl group. However, both the CT26+CJME 10 and CT26+CJME 20 groups showed marked increases in eWAT weight compared to the CT26 group (Figure 3A). Additionally, the CT26 group showed decreased weights of inguinal white adipose tissue (ingWAT) and interscapular brown adipose tissue (iBAT) compared to the Ctrl group. Both CJME-treated groups exhibited significant increases in the weights of ingWAT and iBAT compared to the CT26 group, with no notable differences between the CT26+CJME 10 and CT26+CJME 20 groups (Figures 3B, C). To evaluate adipocyte morphology, we performed H&E staining on eWAT and measured the cross-sectional area (CSA) of adipocytes. The CSA of adipocytes was significantly reduced in the CT26 group compared to the Ctrl group. In contrast, both the CT26+CJME 10 and CT26+CJME 20 groups exhibited an increase in adipocyte CSA compared to the CT26 group (Figure 3D). These findings suggest that CT26-induced cancer cachexia leads to fat tissue loss, and that CJME effectively counteract this loss.

### 3.4 CJME normalizes biochemical parameters related to cancer cachexia and reduces IL-6 production in CT26-induced cancer cachexia model

**Figure 4:**
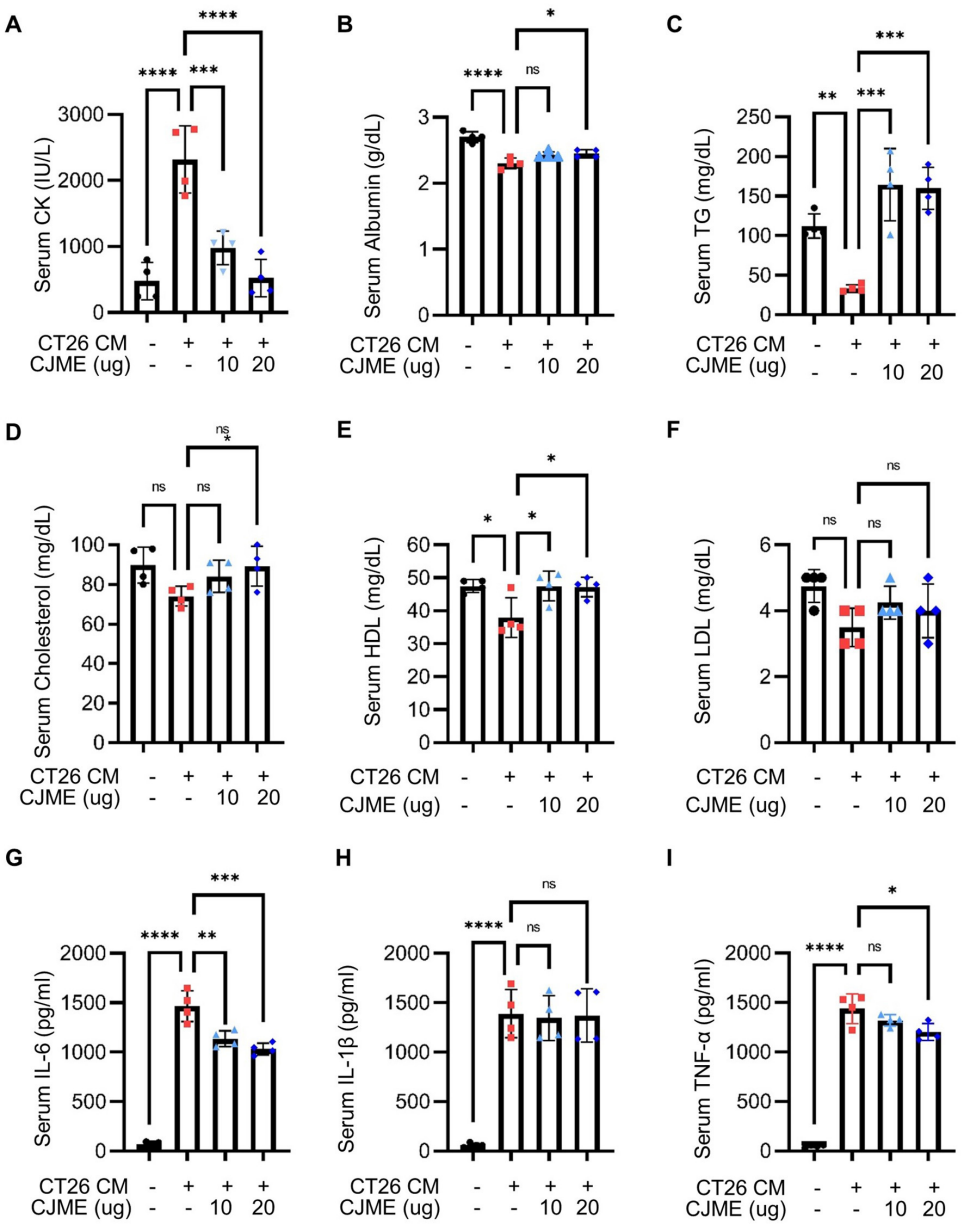
The effect of CJME on biochemical parameters and pro-inflammatory cytokines level in serum of CT26 induced-cancer cachexia model. CT26 cells (2.5 × 10^6^/mouse) were subcutaneously injected into the flank of BALB/c mice, and starting from day 7 post-injection, CJME (10 and 20 mg/kg) was orally administered daily for 1 weeks. The negative control group (Ctrl) was not injected with CT26 cells, and the positive control group (CT26) was orally administered PBS at the same dose as CJME. **(A–F)** Creatine kinase (CK) **(A)**, albumin **(B)**, triglycerides (TG) **(C)**, cholesterol **(D)**, high-density lipoprotein (HDL) **(E)** and low-density lipoprotein (LDL, **F**) levels were measured in serum of CT26 induced mice. **(G–I)** IL-6 **(G)**, IL-1β **(H)** and TNF-α **(I)** levels were measured in serum of CT26 induced mice (*n* = 4 mice per group). Data are representative of either two independent experiments. Data are presented in terms of the mean ± standard deviation and analyzed using a one-wayANOVA or Student's *t*-test. *P* < 0.05 (*), <0.01 (**), <0.001 (***), or <0.0001 (****) were considered statistically significant.

Abnormal serum creatine kinase (CK) levels have been reported in patients with cancer cachexia (35). Additionally, cancer cachexia is associated with decreases in serum albumin, triglycerides (TG), cholesterol, high-density lipoprotein (HDL), and low-density lipoprotein (LDL) and increases in pro-inflammatory cytokines such as IL-6, IL-1β, and TNF-α (17, 36, 37). To investigate these parameters, we isolated serum from a CT26-induced cancer cachexia mouse model to measure the levels of CK, albumin, TG, cholesterol, HDL, LDL, and pro-inflammatory cytokines. The CK level was significantly elevated in the CT26 group compared to the Ctrl group, while a marked decrease in CK levels was observed in the CJME-treated group compared to the CT26 group (Figure 4A). Furthermore, the levels of albumin, TG, cholesterol, and HDL were significantly reduced in the CT26 group compared to the Ctrl group. In contrast, the CJME-treated group demonstrated significant increases in albumin, TG, cholesterol, and HDL levels compared to the CT26 group (Figures 4B–E). The LDL level was significantly lower in the CT26 group compared to the Ctrl group. Although the average LDL level in the CJME administration group increased slightly compared to the CT26 group, there was no statistically significant difference (Figure 4F). Regarding pro-inflammatory cytokines, IL-6 levels were significantly higher in the CT26 group compared to the Ctrl group. Both the CT26+CJME 10 and CT26+CJME 20 groups exhibited reductions in IL-6 levels compared to the CT26 group (Figure 4G). In contrast, while IL-1β level was significantly elevated in the CT26 group compared to the Ctrl group, there was no significant differences between the CJME-treated groups and the CT26 group. However, TNF-α levels were reduced in the CT26+CJME 20 group compared to the CT26 group (Figures 4H, I). These results indicate that CJME effectively restores the abnormal levels of biochemical parameters such as CK, albumin, TG, cholesterol, HDL, and LDL altered by CT26-induced cancer cachexia, and reduce the elevated production of IL-6 and TNF-α.

### 3.5 LC-MS base peak chromatogram of ethanol extracts of *Coptis japonica Makino*

**Figure 5:**
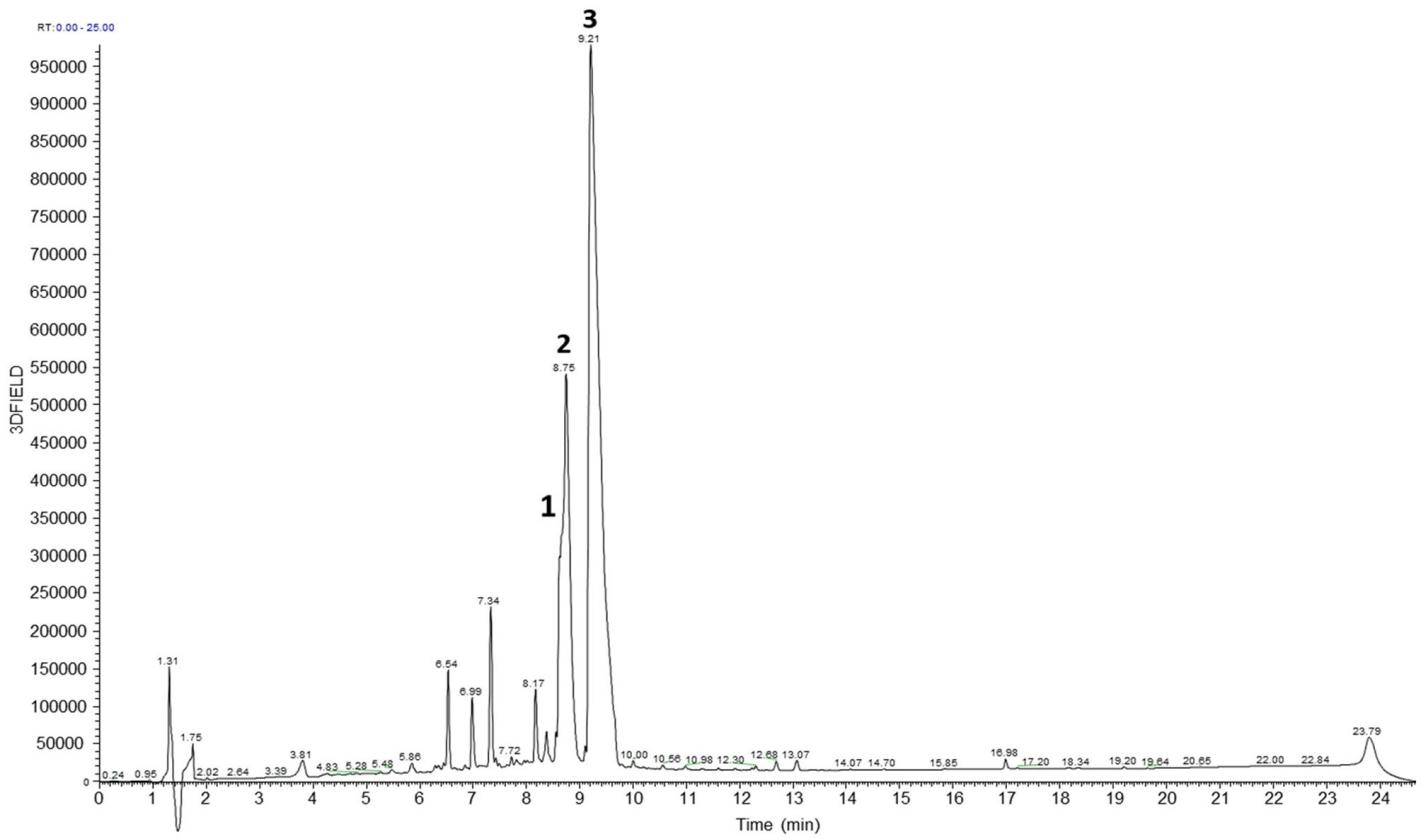
LC-MS profiles of the 70% EtOH extracts of Coptis japonica Makino. Coptisine (1), Palmatine (2), and Berberine (3).

The analysis of 70% ethanol extracts of coptis japonica Makino was conducted using HPLC-mass spectrometry in positive ion mode. By comparing the retention times with those of reference compounds obtained from the plant, three major peaks were identified (Figure 5). The LC-MS chromatogram reveals the presence of three major bioactive compounds in the *Coptis japonica Makino* (CJM) extract: Coptisine, Palmatine, and Berberine. These compounds are characteristic alkaloids known for their anti-inflammatory, antioxidant, and anticancer properties, which are consistent with the therapeutic effects observed in this study. Coptisine is a known alkaloid with significant anti-inflammatory, antibacterial activity and anticancer properties (38–40). Also, palmatine has been reported to possess anti-inflammatory and cytotoxic activities (41, 42), berberine, one of the most studied compounds from CJM, is well-known for its wide-ranging effects, including modulating the immune response and reducing tumor progression, which are crucial in mitigating cancer cachexia (43, 44). The chromatographic data supports the presence of these three compounds, which likely contribute to the observed therapeutic effects of CJM in mitigating cancer cachexia.

## 4 Discussion

Cancer cachexia is a multifaceted syndrome by significant weight loss, muscle and fat depletion, fatigue, and reduced appetite. This condition arises in cancer patients due to tumor growth, metabolic alterations, and the overproduction of inflammatory cytokines (1, 2). As tumors progress, they disrupt the host's metabolism, diminish appetite, and promote the breakdown of muscle and adipose tissues, leading to continuous weight loss, muscle atrophy, and fat depletion. These physiological changes severely impair survival rates and quality of life, leading to poor treatment outcomes and becoming a major cause of death in patients with cancer. In colorectal cancer, cachexia affects an estimated 50–61% of patients (45, 46). Despite ongoing research and clinical trials targeting cancer cachexia, there are still very few approved therapies available. Therefore, it is important to develop safe and effective treatments for patients suffering from this disease.

*Coptis japonica Makino* (CJM) has been traditionally utilized in Asian medicine to treat various ailments, such as intestinal bacterial infections and herpes simplex (24, 47). Recent studies have highlighted additional properties of CJM, such as its anti-inflammatory, antibacterial, and anti-photooxidative effects (22–24). However, despite the existing data on CJM's various effects, there is currently no information regarding the ability of CJM extract (CJME) to inhibit cancer cachexia or any elucidation of its underlying mechanisms. This study aims to provide the first evidence of CJME's inhibitory effects on cancer cachexia through both *in vitro* and *in vivo* experiments.

In our *in vitro* study, we induced atrophy in C2C12 myotubes using CT26 CM and evaluated the effects of CJME. Our results demonstrated that CJME significantly reduced the mRNA and protein levels of the muscle-specific E3 ubiquitin ligases Atrogin-1 and MuRF1, while increasing the protein levels of MyHC. Additionally, CJME notably reversed the atrophy induced by CT26 CM. *In vivo*, CT26-induced cancer cachexia in mice led to significant muscle and fat loss, which CJME effectively reversed. CJME demonstrated the ability to reverse muscle and fat loss caused by CT26-induced cancer cachexia. These findings suggest that CJME has potential as a treatment for cancer cachexia.

Proinflammatory cytokines such as IL-6, IL-1β, TNF-α, and IFN-γ are associated with muscle wasting in both clinical and preclinical cancer cachexia (48). Notably, IL-6 activates the JAK/STAT3 pathway, and the activation of STAT3 is a common feature of muscle wasting conditions (49). STAT3 activation stimulated by IL-6 and various cancers increases the expression of Atrogin-1 and MuRF-1, thereby enhancing the ubiquitin-proteasome system and contributing to muscle degradation (50–53). Our findings demonstrated that CJME inhibits the phosphorylation of STAT3 and reduces IL-6 production in C2C12 myotubes. Furthermore, CJME treatment decreased serum IL-6 levels in mice with the CT26-induced cancer cachexia model. These findings indicate that CJME mechanistically reduces STAT3 phosphorylation, which in turn suppresses the expression of key skeletal muscle atrophy markers such as MuRF-1 and Atrogin-1, while increasing MyHC expression. Therefore, the protective effect of CJME against muscle wasting is at least partially attributable to the inhibition of the STAT3 pathway.

We also examined the impact of cancer cachexia on blood biochemical markers, which are indicative of muscle and fat wasting. Creatine kinase (CK) and albumin serve as valuable indicators of skeletal muscle damage. Serum creatine kinase levels reflect both muscle loss and the extent of muscle damage (14), while serum albumin levels are known to decrease in patients with cancer cachexia (15). Furthermore, triglyceride hydrolysis is a key metabolic pathway implicated in cachexia progression (16). In cachexia patients, levels of total cholesterol, high-density lipoprotein (HDL), and low-density lipoprotein (LDL) typically decrease due to weight loss, metabolic changes, and fat tissue depletion, impacting cholesterol production (17). Our findings revealed that serum CK levels were significantly elevated in the CT26-induced cancer cachexia mice compared to the control group, while CK levels were notably reduced in the CJME-treated groups compared to the CT26 group. Additionally, the CT26 group exhibited significantly decreased levels of albumin, TG, cholesterol, and HDL compared to the Ctrl group. Conversely, the CJME-treated groups showed significant increases in these parameters compared to the CT26 group. These findings suggest that CJME may prevent muscle and fat wasting caused by CT26-induced cancer cachexia by normalizing abnormal biochemical markers such as CK, albumin, TG, cholesterol, HDL, and LDL. However, further studies are needed to elucidate the mechanisms by which CJME affects these biochemical markers and to evaluate whether CK, albumin, TG, cholesterol, HDL, and LDL can serve as clinically important biomarkers.

## 5 Conclusion

In summary, this study provides the first evidence of the inhibitory effect of CJME on cancer cachexia. Our findings demonstrate that CJME attenuated CT26-induced muscle atrophy in C2C12-derived myotubes, which may be associated with the inhibition of STAT3 signaling. Furthermore, administration of CJME in a CT26-induced cancer cachexia mouse model effectively normalized biochemical parameters related to cancer cachexia, suppressed IL-6 production, and improved the overall condition by inhibiting muscle and fat wasting. These findings suggest that CJME is a promising therapeutic candidate for the management of cancer cachexia.

## Data Availability

The raw data supporting the conclusions of this article will be made available by the authors, without undue reservation.
