# Supplementary material for: Coptis japonica Makino ethanol extracts attenuates cancer cachexia induced muscle and fat wasting through inhibition of the STAT3 signaling pathway
Source: Front Nutr. 2025 May 21;12:1509086. doi: 10.3389/fnut.2025.1509086 (PMC12133494; doi:10.3389/fnut.2025.1509086)
Supplement: Supplementary file 1 [file Table_1.docx]

**Supplementary Table 1. Relative muscle weight/body weight (%)**

| **Muscles** | **Control** | **CT26** | **CT26+CJME 10** | **CT26+CJME 20** |
| --- | --- | --- | --- | --- |
| Pectoralis | 0.521±0.065 | 0.323±0.067 | 0.424±0.042 | 0.453±0.054 |
| Triceps | 0.458±0.068 | 0.327±0.046 | 0.417±0.027 | 0.401±0.037 |
| Quadriceps | 0.759±0.145 | 0.534±0.094 | 0.769±0.020 | 0.753±0.086 |
| Tibialis anterior | 0.283±0.048 | 0.256±0.028 | 0.285±0.032 | 0.306±0.030 |
| Gastrocnemius | 0.605±0.080 | 0.494±0.046 | 0.551±0.050 | 0.554±0.058 |
